# Two new freshwater fish species of the genus *Telestes* (Actinopterygii, Cyprinidae) from karst poljes in Eastern Herzegovina and Dubrovnik littoral (Bosnia and Herzegovina and Croatia)

**DOI:** 10.3897/zookeys.180.2127

**Published:** 2012-04-05

**Authors:** Nina G. Bogutskaya, Primož Zupančič, Ivan Bogut, Alexander M. Naseka

**Affiliations:** 1 Zoological Institute, Russian Academy of Sciences, 1 Universitetskaya Emb., St Petersburg 199034, Russia; 2 Dolsko 14, 1262 Slovenia; 3 Agronomski fakultet u Sveučilišta u Mostaru, Zavod za ribarstvo, zoologiju i zaštitu voda, Biskupa Čule 10, 88000 Mostar, Bosnia and Hercegovina

**Keywords:** Cypriniformes, West Balkans, Alpha Taxonomy and new taxa

## Abstract

Two new species, *Telestes dabar* and *Telestes miloradi*, are described on the basis of morphological comparisons of isolated geographical populations of fishes identified earlier as *Telestes metohiensis*. A lectotype is designated for *Telestes metohiensis*, whose range is shown to include waters of Gatačko, Cerničko, and Nevesinjsko poljes in Eastern Herzegovina. *Telestes dabar* from Dabarsko Polje (Eastern Herzegovina) and *Telestes miloradi* from Konavosko Polje (south Croatia) share with *Telestes metohiensis* the following combination of characters that distinguish them from the rest of the genus *Telestes*: pharyngeal teeth in one row, usually 5–4; preoperculo-mandibular canal not communicating with the infraorbital canal; mouth subterminal, the tip of the mouth cleft on or below the level of the ventral margin of the eye; postcleithrum minute or absent; ventral portion of the trunk with a dark stripe on a pale background; and dorsal portion of trunk uniformly dark and bordered ventrally by a dark midlateral stripe. *Telestes dabar* and *Telestes miloradi* are distinguishable from *Telestes metohiensis* in usually having 8½ branched dorsal-fin rays (vs. usually 7½), 9 or 10 gill rakers (vs. 7–10, usually 8), and the dark stripe on the ventral portion of the trunk below the main pigmented area of the back narrow and usually not reaching posteriorly to the caudal peduncle (vs. dark stripe wide and extending posteriorly to the caudal peduncle). *Telestes dabar* is distinguished from *Telestes miloradi* by having scales on most of the body situated close to one another and overlapping in a region behind the pectoral girdle and usually on the caudal peduncle (vs. overlapping scales on most of the body); the lateral line usually incomplete and interrupted, with 24–69, usually 54–65, total scales (vs. lateral line usually complete, with 55–67 total scales); scales above and below the lateral line slightly smaller than lateral-line scales (vs. of about equal size); head width 43–52% HL (vs. 48–58% HL); and lower jaw length 10–12% SL or 36–41% HL (vs. 8–10% SL or 33–38% HL). *Telestes miloradi*, a very local endemic species,is known only by historical samples. *Telestes dabar* is an abundant fish in Dabarsko Polje, but its range is critically restricted during the dry season by a few permanent sources. Nothing is known about its occurrence in underground karst waters.

## Introduction

Karst basins in Croatia and Bosnia and Herzegovina are populated by many local endemic species most of them belonging to the cyprinid subfamily Leuciscinae (Mrakovčić et al. 2006; Kottelat and Freyhof 2007; Jelić et al. 2008; Zupančič 2008). Among leuciscine cyprinids of the Dinaric karst, most numerous is a group of species that was formerly assigned to the genus *Phoxinellus* Heckel, 1843. In a revision of this group (Zupančič and Bogutskaya 2002; Bogutskaya and Zupančič 2003) ten *Phoxinellus* species were divided into two groups, one with two subgroups. Later (Freyhof et al. 2006), based on nuclear and mtDNA sequences, *Phoxinellus* was found to be paraphyletic because it includes three unrelated monophyletic units. The scientific name *Phoxinellus* was therefore restricted to species having plain colouration, a small or absent postcleithrum, no genital papilla, and an almost entirely naked body (species included: type species *Phoxinellus alepidotus* Heckel, 1843, *Phoxinellus dalmaticus* Zupančič & Bogutskaya, 2000, and *Phoxinellus pseudalepidotus* Bogutskaya & Zupančič, 2003). Species having an irregularly spotted colour pattern, a large postcleithrum, an increased number of precaudal anal-fin pterygiophores, and a large female genital papilla were assigned to a new genus, *Delminichthys* Freyhof, Lieckfeldt, Bogutskaya, Pitra & Ludwig, 2006 (species included: type species *Delminichthys adspersus* (Heckel, 1843), *Delminichthys ghetaldii* (Steindachner, 1882), *Delminichthys jadovensis* Zupančič & Bogutskaya, 2002, and *Delminichthys krbavensis* Zupančič & Bogutskaya, 2002). Finally, species having a small or absent postcleithrum, no genital papilla, a dark midlateral stripe from the head to the caudal peduncle, and non-overlapping scales were assigned to *Telestes* (*Telestes croaticus* (Steindachner, 1866), *Telestes fontinalis* (Karaman, 1972), and *Telestes metohiensis* (Steindachner, 1901)), bringing the total number of species of *Telestes* to 11. These generic assignments were later supported by a molecular phylogenetic study based on the same mitochondrial but different nuclear markers (Perea et al. 2010).

Zupančič and Bogutskaya (2002) and Bogutskaya and Zupančič (2003) redescribed *Telestes metohiensis* based on 251 specimens (including syntypes) from Nevesinjsko Polje, Gatačko Polje, Cerničko Polje, and Dabarsko Polje karsts in Bosnia and Herzegovina, and from Ljuta River in Konavle Region [Konavosko Polje] in southern Croatia. Bogutskaya and Zupančič (2003: 375) mentioned that the syntypes from the Ljuta River were distinguished by having 8½ branched anal-fin rays (vs. usually 7½ in most other syntypes). Specimens from Dabarsko Polje (Vrijeka and Opačica rivers) were also only tentatively identified as *metohiensis* because of having overlapping scales (vs. non-overlapping, narrowly to widely spaced) and usually 8½ branched anal-fin rays (Zupančič and Bogutskaya 2002: 417). Since 2003, we have examined additional material of the entire *Telestes metohiensis* species complex, and two species – one from Ljuta River in Konavosko Polje and one from Dabarsko Polje – are described here as new.

## Methods

Measurements were made point to point to the nearest 0.1 mm and follow methods used by Bogutskaya and Zupančič (2010) for *Squalius* species. In most aspects, they coincide with the scheme by Kottelat and Freyhof (2007: fig. 1), but a few clarifications of the latter are as follows. Standard length (SL) was measured from the anteriormost point of the upper lip (not of the snout) to the end of the hypural complex. Head length (HL) was measured from the anteriormost point of the upper lip to the posteriormost point of the opercular membrane. Interorbital width was measured including the skin fold. The term ‘length of dorsal fin’ is used for the length of the dorsal-fin base, and the term ‘depth of dorsal fin’ is used for the length of the longest ray of the dorsal fin. Respective terms are used for the measurements of the anal fin. The last two branched rays articulating on a single pterygiophore in the dorsal and anal fins are noted as “1½”. Total lateral-line scale count includes all pored scales, from the first one just behind the posttemporal bone to the posteriormost one located on the bases of the caudal-fin rays. Total number of lateral-row scales includes all scales, pored and non-pored, from the first one just behind the posttemporal bone to the posteriormost one located on the bases of the caudal-fin rays. Osteological characters were examined from dissections and from radiographs. Statistical analyses were performed with Microsoft Excel and Statistica 6.0 packages.

Abbreviations used: NMW, Naturhistorisches Museum, Wien; SMNH, Slovenian Museum of Natural History; PZC, Collection of P. Zupančič, Dolsko (Slovenia); SMF, Senckenberg Museum, Frankfurt a. Main; ZISP, Zoological Institute, Russian Academy of Sciences, St Petersburg; ZMH, Zoologisches Museum und Institut, Universität Hamburg; (cephalic sensory canals) CIO, infraorbital canal; CPM, preoperculo-mandibular canal; CSO, supraorbital canal; and CST, supratemporal canal.

## Results

### Telestes dabar sp. n.

urn:lsid:zoobank.org:act:4A50A07B-57BD-472B-9B00-6941D8C6779A

http://species-id.net/wiki/Telestes_dabar

Figs 1a 2a 3a

#### Holotype.

NMW 95295, 79.1 mm SL, BOSNIA & HERZEGOVINA: Dabarsko Polje, Opačica River at Potkom, 43°5.9'N, 18°7.6'E, 15 Sept. 2006, coll. Zupančič.

#### Paratypes.

NMW 95300, 6, 55.2–71.1 mm SL, same data as holotype; PZC 525, 72, 32.5–51.3 mm SL, same data as holotype; PZC 526, 13, 48.0–73.8 mm SL, same data as holotype; PZC 565, 21, 35.5–56.7 mm SL, same locality, 8 July 2011; ZISP 54995, 15, 38.1–58.9 mm SL, same locality, 8 July 2011; SMNH 444, 35.5–60.8 mm SL, same locality, 8 July 2011; PZC 279, 12, 44.2–71.9 mm SL, BOSNIA & HERZEGOVINA: Dabarsko Polje, Vrijeka River, 24 May 2001; PZC 521, 13, 54.0–62.6 mm SL, same locality as 279, 15 Sept. 2004; PZC 575, 18, 40.5–69.8 mm SL, same locality as 279, 15 Sept. 2006.

#### Diagnosis.

*Telestes dabar* is distinguished from *Telestes metohiensis* and *Telestes miloradi* by having the following combination of characters: slightly curved dark stripe (obvious in live and preserved specimens) present from just behind operculum to vertical just anterior to origin of anal fin, this stripe narrow and separated from dark pigmented area on back along its entire length; scales on most of body not overlapping but situated close to one another; scales overlapping behind pectoral girdle along lateral line and usually on caudal peduncle; snout with fleshy tip projecting over upper lip; mouth subterminal with tip of mouth cleft at or below level of ventral margin of eye; lateral line usually interrupted, with 24–69 total lateral-line scales; branched dorsal-fin rays usually 8½; branched anal-fin rays usually 8½; gill rakers 9 or 10; total vertebrae 39–41, mode 40; abdominal vertebrae 22–24, mode 22; caudal vertebrae 16–18, mode 17; head width 43–52% HL; and lower jaw long, length 10–12% SL.

#### Description.

**Figure 1.**
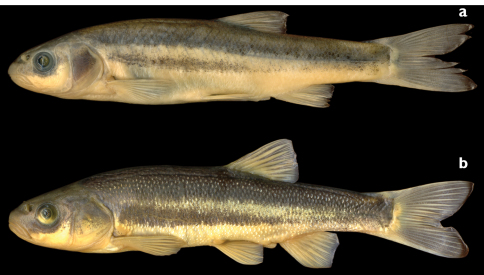
**a** *Telestes dabar*, holotype, female, 79.1 mm SL, NMW 95295, Bosnia & Herzegovina: Opačica River, Dabarsko Polje **b** *Telestes metohiensis*, female, 82.1 mm SL, PZC 293, Bosnia & Herzegovina: Zovidolka River (Zalomka River system), Nevesinjsko Polje.

**Figure 2.**
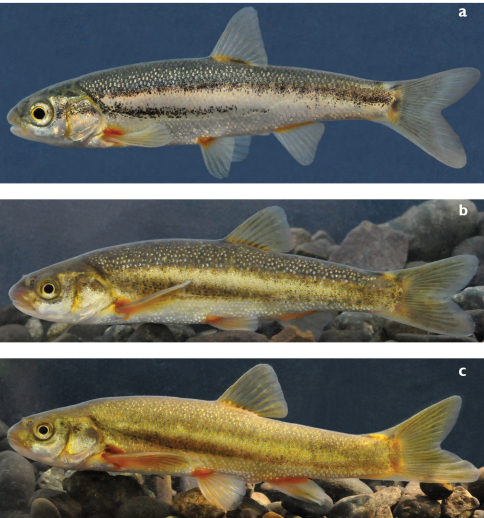
**a** *Telestes dabar*, male paratype, live specimen, ZISP 54995, 58.9 mm SL, Bosnia & Herzegovina: Opačica River, Dabarsko Polje **b** *Telestes metohiensis*, live specimen, male, 86.2 mm SL, PZC 567, Bosnia & Herzegovina: spring Ljeskovik in Zalomka River, Nevesinjsko Polje **c** *Telestes metohiensis*, live specimen, male, 84.5 mm SL, PZC 566, Bosnia & Herzegovina: Zovidolka River (Zalomka River system), Nevesinjsko Polje.

Morphometric data are summarised in Table 1a, selected counts in Tables 2–4. General appearance can be seen in Figs 1a and 2a. Body compressed, elongate. Caudal-peduncle depth only slightly less than half maximum body depth; head length greater than maximum body depth. Eye small, its diameter smaller than snout length. Snout fleshy, slightly to markedly projecting beyond upper lip (similar to a feature Kottelat and Freyhof [2007: fig. 39] called the “rostral cap,” which covers all or part of upper lip); snout terminating laterally in prominent crease along anterior edge of first infraorbital. Mouth subterminal, tip of mouth cleft at level of ventral margin of eye or, more frequently, below it. Lower jaw-quadrate junction at vertical through anterior half of eye. Length of lower jaw 10–12% SL or 36–41% HL, or 102–132% depth of operculum.

**Table 1a.** Morphometric data of *Telestes dabar*.

	*Telestes dabar*								
	Holotype	females, n=26				males, n=13			
		min	max	mean	SD	min	max	M	SD
SL, mm	79.1	60.3	81.8	67.8		55.5	69.0	61.1	
Maximum body depth (% SL)	21.6	21.6	26.0	23.6	1.3	21.7	25.0	22.6	0.9
Depth of caudal peduncle (% SL)	9.8	9.8	12.4	11.0	0.7	10.0	12.4	11.2	0.7
Depth of caudal peduncle (% length of caudal peduncle)	47.9	47.7	59.6	53.6	4.3	44.6	62.2	52.9	5.2
Maximum body width (% SL)	13.4	11.4	16.0	13.7	1.2	12.0	22.0	14.2	2.6
Predorsal length (% SL)	55.3	53.5	58.7	56.9	1.1	53.8	57.3	55.5	1.2
Postdorsal length (% SL)	36.3	32.8	36.6	35.2	1.0	34.4	37.2	35.8	1.0
Prepelvic length (% SL)	51.6	50.4	55.5	52.7	1.4	50.6	54.4	52.2	1.0
Preanal length (% SL)	71.2	68.3	73.2	70.8	1.3	68.6	71.9	69.9	1.0
Pectoral – pelvic-fin origin length (% SL)	24.5	24.5	27.7	25.6	0.9	22.3	26.5	24.1	1.2
Pelvic – anal-fin origin length (% SL)	20.2	17.2	20.2	19.0	0.6	16.6	20.3	18.4	1.3
Length of caudal peduncle (% SL)	20.6	18.5	21.9	20.5	0.8	19.2	23.5	21.2	1.1
Dorsal-fin base length (% SL)	10.3	9.3	12.3	10.9	0.8	9.9	12.2	11.2	0.7
Dorsal fin depth (% SL)	18.9	16.6	20.2	18.6	1.4	18.5	21.8	19.7	0.9
Anal-fin base length (% SL)	12.1	9.2	12.1	10.6	0.8	10.3	12.4	11.2	0.5
Anal fin depth (% SL)	12.3	11.9	14.8	13.2	0.8	13.4	17.2	15.9	1.1
Pectoral fin length (% SL)	19.9	18.1	21.2	19.9	0.8	22.5	26.7	24.2	1.3
Pelvic fin length (% SL)	14.8	12.9	16.3	14.9	0.9	16.3	17.7	16.9	0.5
Head length (% SL)	27.1	25.6	28.7	27.1	0.9	26.6	29.6	28.1	1.0
Head length (% body depth)	125.1	104.0	125.1	115.3	5.9	110.8	135.1	124.3	7.6
Head depth at nape (% SL)	17.4	17.1	19.3	18.1	0.7	17.0	18.8	18.2	0.5
Head depth at nape (% HL)	64.2	62.4	70.7	66.8	2.3	60.6	69.3	65.0	2.7
Maximum head width (% SL)	13.5	11.6	14.6	13.6	0.7	12.4	14.5	13.6	0.6
Maximum head width (% HL)	50.0	42.8	52.3	50.1	2.4	44.7	52.1	48.3	2.4
Maximum cranial width (% cranium roof length)	68.9	61.9	72.6	65.8	3.1	59.7	68.8	64.4	2.5
Snout length (% SL)	8.5	7.4	9.2	8.1	0.5	7.8	9.0	8.5	0.4
Snout length (% HL)	31.4	27.7	32.8	29.7	1.3	28.5	31.7	30.3	1.1
Eye horizontal diameter (% SL)	6.6	5.9	7.2	6.6	0.4	6.3	7.8	6.8	0.4
Eye horizontal diameter (% HL)	24.4	22.1	26.8	24.5	1.5	22.1	26.8	24.3	1.4
Eye horizontal diameter (% interorbital width)	74.5	66.3	81.9	73.3	4.7	69.5	82.0	74.4	4.7
Postorbital distance (% HL)	53.0	48.8	54.0	52.2	1.4	47.1	53.0	50.2	1.9
Interorbital width (% SL)	8.9	8.2	9.6	9.1	0.4	8.5	9.9	9.2	0.4
Interorbital width (% HL)	32.8	31.3	36.0	33.4	1.2	29.0	35.6	32.7	1.9
Length of upper jaw (% HL)	28.6	24.5	30.5	27.8	1.9	26.2	29.5	27.7	1.1
Length of upper jaw (% SL)	7.7	6.6	8.5	7.5	0.6	7.0	8.6	7.8	0.5
Length of lower jaw (% SL)	10.6	9.5	11.2	10.2	0.5	9.7	11.5	10.9	0.5
Length of lower jaw (% HL)	39.2	35.8	40.7	37.6	1.3	35.6	41.1	38.6	1.6
Length of lower jaw (% interorbital width)	119.7	106.4	123.4	112.6	4.4	110.2	129.8	120.2	6.4
Length of lower jaw (% depth of operculum)	110.2	102.4	120.0	108.2	4.5	105.7	131.7	116.6	8.2

**Table 1b.** Morphometric data of *Telestes miloradi*.

	*Telestes miloradi*								
	Holotype	females, n=9				males, n=3			
		min	max	mean	SD	min	max	Mean	SD
SL, mm	66.7	34.1	66.7			57.9	61.8		
Maximum body depth (% SL)	22.4	21.7	25.8	23.5	1.5	19.6	24.6	23.0	2.9
Depth of caudal peduncle (% SL)	11.4	10.4	11.6	11.2	0.4	9.6	11.3	10.8	1.0
Depth of caudal peduncle (% length of caudal peduncle)	53.8	49.8	54.8	52.4	2.1	41.8	52.8	49.1	6.4
Maximum body width (% SL)	14.3	12.9	15.3	14.6	0.7	11.4	16.8	15.0	3.1
Predorsal length (% SL)	55.9	55.9	57.7	57.0	0.7	54.3	54.9	54.5	0.3
Postdorsal length (% SL)	35.2	32.8	35.3	34.3	1.0	35.4	35.6	35.5	0.1
Prepelvic length (% SL)	51.6	51.5	53.3	52.3	0.9	50.0	50.2	50.1	0.1
Preanal length (% SL)	72.8	68.2	72.8	70.3	1.9	68.2	68.4	68.3	0.1
Pectoral – pelvic-fin origin length (% SL)	25.1	25.1	27.6	26.2	1.1	23.3	25.0	24.4	1.0
Pelvic – anal-fin origin length (% SL)	20.8	16.6	20.8	18.3	1.7	17.6	18.4	18.2	0.5
Length of caudal peduncle (% SL)	21.2	20.5	22.1	21.3	0.7	21.4	23.0	22.0	0.9
Dorsal-fin base length (% SL)	11.6	9.9	11.6	10.9	0.7	11.2	12.6	12.2	0.8
Dorsal fin depth (% SL)	19.6	19.2	20.9	19.8	0.8	20.9	22.1	21.4	0.6
Anal-fin base length (% SL)	12.4	10.7	12.4	11.9	0.6	11.8	11.9	11.8	0.1
Anal fin depth (% SL)	13.2	0.0	15.0	12.5	4.7	13.7	15.5	14.3	1.0
Pectoral fin length (% SL)	21.7	18.5	21.7	20.8	1.0	23.5	25.4	24.8	1.1
Pelvic fin length (% SL)	15.6	13.6	15.6	14.6	0.8	16.9	17.6	17.2	0.4
Head length (% SL)	26.8	25.4	28.0	27.0	0.8	26.5	29.0	28.2	1.4
Head length (% body depth)	119.9	105.0	119.9	115.0	6.0	117.6	135.4	123.5	10.3
Head depth at nape (% SL)	18.3	16.9	18.3	17.6	0.5	16.7	19.0	18.2	1.4
Head depth at nape (% HL)	68.1	62.5	68.1	65.1	2.3	62.9	65.7	64.7	1.6
Maximum head width (% SL)	14.1	12.3	14.5	14.0	0.7	12.6	16.9	15.4	2.5
Maximum head width (% HL)	52.5	48.7	53.6	52.0	1.5	47.5	58.2	54.6	6.2
Maximum cranial width (% cranium roof length)	62.8	59.6	73.9	66.0	5.0	58.3	65.2	62.9	4.0
Snout length (% SL)	9.0	8.0	9.7	8.9	0.5	8.5	8.6	8.6	0.0
Snout length (% HL)	33.7	31.4	34.7	33.0	1.2	29.5	32.4	30.5	1.7
Eye horizontal diameter (% SL)	6.5	6.3	6.9	6.5	0.2	6.6	6.7	6.7	0.0
Eye horizontal diameter (% HL)	24.4	22.5	26.4	24.2	1.4	22.9	25.2	23.7	1.3
Eye horizontal diameter (% interorbital width)	70.2	64.7	83.0	71.3	5.9	69.4	83.4	74.1	8.1
Postorbital distance (% HL)	49.1	48.9	51.6	50.2	1.2	50.2	51.0	50.5	0.5
Interorbital width (% SL)	9.3	8.1	9.7	9.2	0.5	8.0	9.6	9.1	0.9
Interorbital width (% HL)	34.8	31.9	34.8	34.1	0.9	30.2	33.1	32.1	1.7
Length of upper jaw (% HL)	27.9	26.0	28.1	27.2	0.9	23.5	25.1	24.0	0.9
Length of upper jaw (% SL)	7.5	6.6	7.9	7.3	0.4	6.7	6.8	6.8	0.1
Length of lower jaw (% SL)	9.3	8.4	10.4	9.5	0.6	8.7	10.4	9.8	1.0
Length of lower jaw (% HL)	34.7	33.1	38.4	35.3	1.8	32.9	35.9	34.9	1.7
Length of lower jaw (% interorbital width)	99.8	99.8	114.3	103.7	6.1	108.5	109.1	108.7	0.4
Length of lower jaw (% depth of operculum)	104.0	95.6	104.0	101.1	2.9	95.6	107.1	103.3	6.7

**Table 1c.** Morphometric data of *Telestes metohiensis*.

	*Telestes metohiensis*, Gatačko and Cerničko poljes								
	lectotype	females, n=25				males, n=10			
		min	max	mean	SD	min	max	mean	SD
SL, mm	87.9	60.26	102.1	74.9		54.4	75.7	63.1	
Maximum body depth (% SL)	23.9	19.9	25.1	22.5	1.4	20.7	24.1	22.5	1.0
Depth of caudal peduncle (% SL)	11.3	9.7	11.9	10.7	0.5	10.4	11.8	11.2	0.5
Depth of caudal peduncle (% length of caudal peduncle)	61.5	46.7	61.5	53.0	4.1	46.7	55.7	51.0	3.1
Maximum body width (% SL)	16.0	11.1	16.4	14.3	1.7	14.1	15.6	14.8	0.6
Predorsal length (% SL)	57.6	49.4	60.1	56.5	2.2	53.7	57.7	55.7	1.3
Postdorsal length (% SL)	33.4	31.0	36.5	33.7	1.6	32.5	38.0	35.2	1.6
Prepelvic length (% SL)	52.0	46.0	55.9	52.7	1.9	50.4	53.8	51.9	1.3
Preanal length (% SL)	69.1	65.6	73.6	70.2	1.9	67.1	69.7	68.5	0.9
Pectoral – pelvic-fin origin length (% SL)	27.5	24.7	29.7	27.0	1.3	24.1	27.2	25.5	1.1
Pelvic – anal-fin origin length (% SL)	19.8	16.4	20.1	18.5	1.1	15.6	18.2	17.0	0.8
Length of caudal peduncle (% SL)	18.3	19.1	22.3	20.3	1.2	20.9	22.8	22.0	0.6
Dorsal-fin base length (% SL)	11.1	9.5	11.5	10.8	0.6	10.8	13.6	12.0	1.0
Dorsal fin depth (% SL)	17.6	14.9	18.3	17.2	0.8	16.3	19.6	18.5	1.1
Anal-fin base length (% SL)	11.3	9.1	12.0	10.9	0.7	10.0	13.2	11.9	1.1
Anal fin depth (% SL)	12.3	12.4	15.3	13.7	0.8	12.8	15.4	14.3	1.0
Pectoral fin length (% SL)	18.4	17.0	19.8	18.4	0.8	22.7	25.3	23.6	0.8
Pelvic fin length (% SL)	12.9	12.4	15.3	13.7	0.8	15.4	17.7	16.6	0.7
Head length (% SL)	27.0	23.8	28.2	26.6	1.1	26.1	28.2	27.3	0.6
Head length (% body depth)	112.9	111.0	132.5	120.4	6.6	114.4	131.8	122.8	5.2
Head depth at nape (% SL)	17.4	15.5	18.7	17.3	0.8	15.9	18.1	17.3	0.7
Head depth at nape (% HL)	64.6	62.9	71.8	65.1	2.2	58.5	66.1	63.5	2.7
Maximum head width (% SL)	14.8	12.9	15.7	14.8	0.7	14.5	15.7	14.9	0.4
Maximum head width (% HL)	54.9	50.6	59.3	55.7	2.7	52.8	56.9	54.7	1.6
Maximum cranial width (% cranium roof length)	63.5	62.9	78.6	68.6	4.4	65.4	71.7	68.2	2.7
Snout length (% SL)	8.1	7.3	8.8	8.2	0.4	8.5	9.4	8.9	0.4
Snout length (% HL)	30.0	27.9	33.1	30.8	1.6	31.2	34.2	32.5	1.1
Eye horizontal diameter (% SL)	6.0	4.6	6.9	5.9	0.5	5.3	6.8	6.0	0.4
Eye horizontal diameter (% HL)	22.4	19.4	25.2	22.1	1.5	19.5	24.8	22.1	1.8
Eye horizontal diameter (% interorbital width)	70.7	57.0	80.2	68.2	6.9	60.2	71.8	65.1	4.3
Postorbital distance (% HL)	51.9	47.9	55.6	51.9	2.4	46.2	53.1	50.8	2.3
Interorbital width (% SL)	8.5	7.7	9.2	8.7	0.4	8.4	9.8	9.3	0.5
Interorbital width (% HL)	31.6	29.6	34.8	32.6	1.7	30.8	35.8	34.0	1.5
Length of upper jaw (% HL)	27.8	27.0	31.0	29.2	1.0	27.0	30.9	29.0	1.1
Length of upper jaw (% SL)	7.5	6.4	8.3	7.8	0.4	7.5	8.4	7.9	0.3
Length of lower jaw (% SL)	9.9	8.3	11.3	10.3	0.8	9.5	11.0	10.3	0.6
Length of lower jaw (% HL)	36.7	35.0	42.2	38.7	2.2	34.8	41.2	37.8	2.1
Length of lower jaw (% interorbital width)	116.0	106.5	132.5	120.6	6.4	101.6	121.9	113.7	6.9
Length of lower jaw (% depth of operculum)	107.2	106.5	117.9	112.5	3.0	109.6	119.2	112.7	3.5

**Table 1d.** Morphometric data of *Telestes metohiensis*.

	*Telestes metohiensis* (*affinis* nomen museale), Nevesinjsko Polje								
	lectotype	females, n=33				males, n=14			
		min	max	mean	SD	min	max	M	sd
SL, mm	87.9	57.8	113.8	79.5		57.7	86.2	70.4	
Maximum body depth (% SL)	23.9	19.3	24.1	21.8	1.1	19.8	23.8	21.6	1.4
Depth of caudal peduncle (% SL)	11.3	9.0	12.0	10.7	0.7	9.6	12.3	11.1	0.7
Depth of caudal peduncle (% length of caudal peduncle)	61.5	42.7	60.0	54.2	4.5	43.6	59.9	51.8	4.2
Maximum body width (% SL)	16.0	11.6	17.6	14.5	1.4	11.0	16.6	14.3	1.7
Predorsal length (% SL)	57.6	54.2	59.4	57.1	1.3	54.4	58.4	56.3	1.0
Postdorsal length (% SL)	33.4	29.9	36.4	34.0	1.3	33.8	36.5	35.2	0.7
Prepelvic length (% SL)	52.0	51.6	55.1	53.3	1.0	50.1	54.6	51.6	1.2
Preanal length (% SL)	69.1	67.8	73.4	70.9	1.3	65.9	70.3	68.8	1.3
Pectoral – pelvic-fin origin length (% SL)	27.5	24.2	30.0	25.9	1.2	21.7	25.4	23.8	1.0
Pelvic – anal-fin origin length (% SL)	19.8	16.7	44.1	19.4	4.6	15.6	19.9	17.5	1.3
Length of caudal peduncle (% SL)	18.3	18.0	21.3	19.7	0.9	20.0	23.2	21.4	0.8
Dorsal-fin base length (% SL)	11.1	9.4	12.9	11.1	0.8	8.1	12.1	10.4	1.2
Dorsal fin depth (% SL)	17.6	15.8	20.4	17.9	1.4	15.9	21.8	18.0	1.5
Anal-fin base length (% SL)	11.3	9.4	12.0	10.9	0.6	10.5	13.2	11.4	0.7
Anal fin depth (% SL)	12.3	10.6	15.0	13.1	1.1	12.0	16.2	14.0	1.4
Pectoral fin length (% SL)	18.4	18.0	21.9	20.2	1.0	21.8	26.4	23.8	1.4
Pelvic fin length (% SL)	12.9	12.5	15.6	14.1	0.8	14.2	17.5	15.7	0.9
Head length (% SL)	27.0	26.7	29.4	27.8	0.8	26.3	30.1	28.0	1.1
Head length (% body depth)	112.9	114.5	142.6	127.4	6.6	116.5	142.9	130.0	8.5
Head depth at nape (% SL)	17.4	16.5	18.7	17.6	0.6	15.7	18.8	17.3	0.9
Head depth at nape (% HL)	64.6	60.0	67.9	63.4	1.8	57.1	65.9	62.0	2.9
Maximum head width (% SL)	14.8	13.7	16.6	14.9	0.6	13.3	16.2	14.7	0.8
Maximum head width (% HL)	54.9	50.5	61.2	53.7	2.3	51.0	57.4	53.0	2.4
Maximum cranial width (% cranium roof length)	63.5	64.9	79.6	72.5	4.3	66.3	75.8	69.1	2.8
Snout length (% SL)	8.1	7.9	9.8	8.7	0.5	7.7	9.3	8.6	0.4
Snout length (% HL)	30.0	29.4	33.4	31.3	1.1	27.2	33.0	30.8	1.6
Eye horizontal diameter (% SL)	6.0	4.7	7.7	6.1	0.7	5.4	8.0	6.3	0.7
Eye horizontal diameter (% HL)	22.4	17.3	27.3	21.9	2.5	20.6	26.4	22.5	2.0
Eye horizontal diameter (% interorbital width)	70.7	52.0	86.7	67.5	8.3	59.8	92.5	73.1	11.9
Postorbital distance (% HL)	51.9	49.7	57.2	53.3	1.9	48.1	54.4	51.2	1.8
Interorbital width (% SL)	8.5	8.0	10.0	9.0	0.6	7.5	9.9	8.7	0.8
Interorbital width (% HL)	31.6	29.7	35.9	32.5	1.9	26.2	34.8	31.2	2.7
Length of upper jaw (% HL)	27.8	26.7	30.5	28.7	0.9	27.4	29.8	28.5	0.8
Length of upper jaw (% SL)	7.5	7.2	8.7	8.0	0.4	7.3	8.9	8.0	0.5
Length of lower jaw (% SL)	9.9	10.0	12.2	10.7	0.5	10.0	12.5	11.0	0.9
Length of lower jaw (% HL)	36.7	36.3	43.8	38.5	1.5	36.3	44.0	38.9	2.5
Length of lower jaw (% interorbital width)	116.0	110.0	133.8	121.2	6.0	109.1	138.7	123.0	9.8
Length of lower jaw (% depth of operculum)	116.5	110.8	133.8	116.1	4.9	111.5	132.3	118.5	7.0

**Figure 3.**
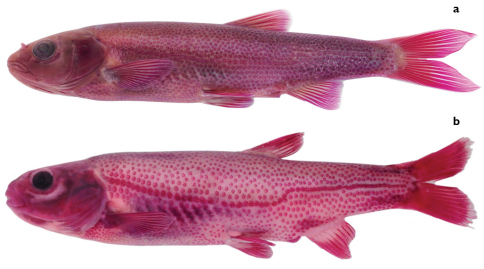
**a** *Telestes dabar*, alizarin stained specimen, PZC 575, 63.7 mm SL, Bosnia & Herzegovina: Vrijeka River, Dabarsko Polje **b** *Telestes metohiensis*, alizarin stained specimen, 63.3 mm SL, PZC 312, Bosnia & Herzegovina: spring Ljeskovik in Zalomka River, Nevesinjsko Polje.

Dorsal fin with 7½ (9 specimens), 8½ (151) or 9½ (1) branched rays, 8½ in holotype. Dorsal-fin origin above posterior end of pelvic-fin base. Anal fin with 8½ (153) or 9½ (8) branched rays, 8½ in holotype. Outer margin of anal fin slightly concave or almost straight. Caudal fin moderately forked, lobes weakly pointed, with 9+8 principal branched rays.

Total gill rakers (Table 2) 9 (20) or 10 (20), 10 in holotype. Pharyngeal teeth 5–4, hooked, slightly serrated (examined in 5 specimens).

**Table 2.** Gill-raker counts in *Telestes dabar*, *Telestes miloradi* and *Telestes metohiensis*.

	7	8	9	10	mean	SD
*Telestes dabar*			20	20	9.5	0.51
*Telestes miloradi*		1	11	6	9.3	0.57
*Telestes metohiensis*, Gatačko Polje	2	33	15	1	8.3	0.58
*Telestes metohiensis*, Nevesinjsko Polje	6	28	12	1	8.2	0.67

Scales covering entire body including pre-pectoral area and abdomen, non-overlapping on most parts of body but overlapping in triangular-shaped area just behind pectoral girdle and usually on caudal peduncle at least behind anal fin (Fig. 3a); lateral-line scales always overlapping, sometimes a few posteriormost scales not overlapping. Scales irregularly set but close to one another. Most flank scales oval, somewhat deeper than long; scales on caudal peduncle more elongated (longer than deep) having prominent posterior attenuation. Trunk scales smaller than lateral-line scales but not considerably so.All scales well ossified, usuallyvisible without staining. In live specimens, scales clearly visible because of some silver highlights (Fig. 2a). Lateral line complete (2 specimens), long but incomplete (5) or interrupted (33) as in specimen in Fig. 3a; if interrupted, gaps typically comprising absence of a few scales in a few places, 24–69 in total (Table 3), 65 with one interruption in holotype. Lateral line making clear curvature above anal-fin origin. Number of scales in total lateral series 62–69 (modal range 65–67), 68 in holotype.

**Table 3.** Total lateral-line scale and total lateral-series scales counts in *Telestes dabar*, *Telestes miloradi* and *Telestes metohiensis*.

	Total lateral-line scales								Total lateral-series scales		
	0–53	54–57	58–61	62–65	66–69	70–73	mean	SD	range	mean	SD
*Telestes dabar*	5	17	8	7	3		54.2	9.58	62–69	66.6	2.41
*Telestes miloradi*		6	5	5	2		60.3	3.91	58–69	62.8	2.93
*Telestes metohiensis*, Gatačko Polje		11	10	25	5		61.0	3.92	56–71	63.5	3.74
*Telestes metohiensis*, Nevesinjsko Polje	1	9	11	19	5	2	61.1	5.32	60–71	65.0	3.07

Parietal segment of CSO lacking. CPM not communicating with CIO, terminating over the upper margin of opercular antedorsal process. CSO complete with 8, rarely 7 or 9, pores. CIO complete with 14–17 pores and with 4 canal openings on first infraorbital. CPM complete or interrupted between the angulo-articular and preoperculum and/or between preoperculum and operculum, CPM with 14–17 pores (4, rarely 5, canal openings on dentary, and 7–9, usually 8, canal openings on preoperculum). CST complete, with 5–7 pores or narrowly interrupted in middle.

Total vertebrae (Table 4) 39 (49), 40 (101) or 41 (11), 40 in holotype; abdominal vertebrae 22 (101), 23 (58) or 24 (2), 22 in holotype; caudal vertebrae 16 (9), 17 (82) or 18 (70), 18 in holotype; predorsal vertebrae 13 (24), 14 (126) or 15 (11), 14 in holotype; intermediate vertebrae 3 (123) or 4 (38), 3 in holotype. Most frequent vertebral formulae 22+17 (41), 22+18 (60) and 23+17 (40), 22+18 in holotype.

**Table 4a.** Vertebral counts in *Telestes* species endemic in Croatia and Bosnia and Herzegovina. Total vertebrae.

	38	39	40	41	42	mean	SD
*Telestes dabar*		49	101	11		39.8	0.56
*Telestes miloradi*		1	10	11		40.5	0.60
*Telestes metohiensis*, Gatačko Polje	6	33	12	2		39.2	0.68
*Telestes metohiensis*, Nevesinjsko Polje	10	112	35			39.2	0.52
*Telestes croaticus*	5	21				38.8	0.40
*Telestes fontinalis*	1	7	3			39.2	0.60
*Telestes karsticus*			2	2	3	41.1	0.89
*Telestes polylepis*			2	9	6	41.2	0.66
*Telestes turskyi*		2	9	6	1	40.3	0.77
*Telestes ukliva*					8	42.0	0

**Table 4b.** Vertebral counts in *Telestes* species endemic in Croatia and Bosnia and Herzegovina. Vertebral formulae.

	21+17	22+16	22+17	22+18	22+19	23+16	23+17	23+18	23+19	24+16	24+17	24+18	24+19
*Telestes dabar*			41	60		8	40	10		1	1		
*Telestes miloradi*			1	6	1		4	10					
*Telestes metohiensis*, Gatačko Polje		6	28	2		5	10	2					
*Telestes metohiensis*, Nevesinjsko Polje		7	58	12		33	24						
*Telestes croaticus*	2	3	16			5							
*Telestes fontinalis*		1	1			7	3						
*Telestes karsticus*				1			1	2	1			2	
*Telestes polylepis*				1			1	8	4		1	2	
*Telestes turskyi*			1	4		1	5	6	1				
*Telestes ukliva*									3				5

**Table 4c.** Vertebral counts in *Telestes* species endemic in Croatia and Bosnia and Herzegovina. Abdominal, caudal and predorsal vertebrae in *Telestes dabar*, *Telestes miloradi* and *Telestes metohiensis*.

	Abdominal vertebrae						Caudal vertebrae							Predorsal vertebrae					
	21	22	23	24	Mean	SD	15	16	17	18	19	mean	SD	12	13	14	15	mean	SD
*Telestes dabar*		101	58	2	22.4	0.51		9	82	70		17.4	0.59		24	126	11	13.9	0.46
*Telestes miloradi*		8	14		22.6	0.49			5	16	1	17.8	0.50			15	7	14.3	0.5
*Telestes metohiensis*, Gatačko Polje		36	17		22.3	0.47		7	42	4		16.9	0.46		3	38	12	14.2	0.50
*Telestes metohiensis*, Nevesinjsko Polje	1	92	64		22.4	0.51	2	43	105	7		16.7	0.56		2	124	31	14.2	0.42
*Telestes croaticus*	2	19	5		22.1	0.52		8	18			16.7	0.47			21	5	14.2	0.40
*Telestes fontinalis*		2	9		22.8	0.40		7	4			16.4	0.50			2	9	14.8	0.40
*Telestes karsticus*		1	4	2	23.1	0.69			1	5	1	18.0	0.58		4	3		13.4	0.53
*Telestes polylepis*		1	13	3	23.1	0.49			2	11	4	18.1	0.60		1	16		13.9	0.24
*Telestes turskyi*		5	13		22.7	0.46		1	6	10	1	17.6	0.62		16	2		13.1	0.32
*Telestes ukliva*			3	5	23.6	0.52				5	3	18.4	0.52	2	5	1		12.9	0.64

#### Colouration.

In live specimens, dark back contrasting sharply with pale area below lateral midline, even in small specimens. Black midlateral stripe extending from head to caudal peduncle forming ventral border of darkly pigmented area on back. Another black lateral stripe occurring more ventrally, on otherwise pale ventral portion of trunk; this stripe extending from eye or opercle (or just behind opercle) to at least vertical through point halfway between origins of pelvic and anal fins, sometimes extending as poorly coalesced spots onto caudal peduncle. Dash-like black marking present along internal procurrent rays of caudal-fin dorsal lobe, and elongate black blotch present at bases of 3rd–7^th^ branched rays of dorsal fin. Black pigment also occurring on rays of dorsal and caudal fins, but its intensity varying among individuals. Peritoneum black. This general pattern of pigmentation retained in formaldehyde-fixed and ethanol-preserved specimens. Live specimens collected from May through September, both males and females, exhibiting yellowish-orange pigment at bases of all fins, especially pectoral and anal fins, and yellowish pigment on iris and along anterior, dorsal and posterior margins of operculum. Colouration of specimens in cold season unknown.

#### Sexual dimorphism.

Genital papilla absent in both males and females. Most morphometric characters not significantly different between males and females (Table 1a) with five exceptions. In males, distance between origins of pectoral and pelvic fins longer than in females (P<0.0001), dorsal fin deeper (P<0.02), anal fin deeper (P<0.0001), pectoral fin longer (P<0.0001), pectoral fin often reaching pelvic-fin origin in males, and pelvic fin longer (P<0.0001), pelvic fin often reaching anal-fin origin in males.

In samples collected in May, ripe males with small but prominent conical breeding tubercles. Tubercles regularly covering entire body, including dorsal and ventral surfaces of caudal peduncle, except for ventralmost surface of head. Single tubercle located on each scale. On all fins (except for caudal fin), tubercles present on both sides along all rays and on fin membrane, being particularly dense along marginal rays. Tubercles forming rows along outer margins of operculum and pectoral fin; tubercles in those rows larger than others on body. Degree of tubercle development varying between males with regard to both size of tubercles and their location. Tubercles always present on head, back, and pectoral fin. Males retaining tubercles, though reduced in size and density, until September.

#### Distribution.

**Figure 4.**
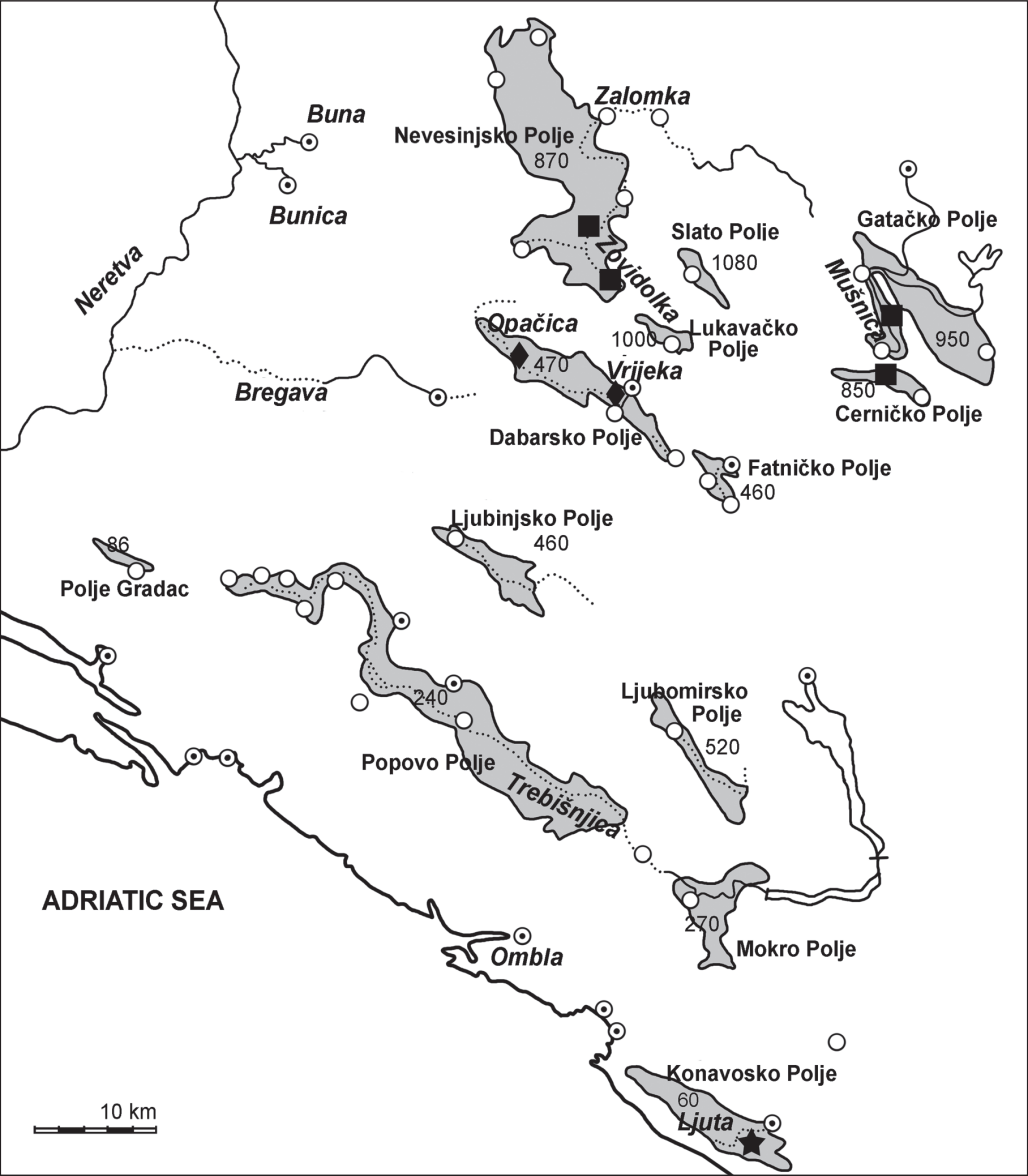
Map of distribution of *Telestes dabar* (diamond)*, T. miloradi* (star) and *Telestes metohiensis* (square)in karst fields of Eastern Herzegovina and Dubrovnik littoral; white circles show ponors and white circles with black dots - springs.

The new species is known from two rivers, Vrijeka and Opačica, in the Dabarsko Polje of Eastern Herzegovina in Bosnia and Herzegovina (Fig. 4).

#### Habitat and biology.

**Figure 5.**
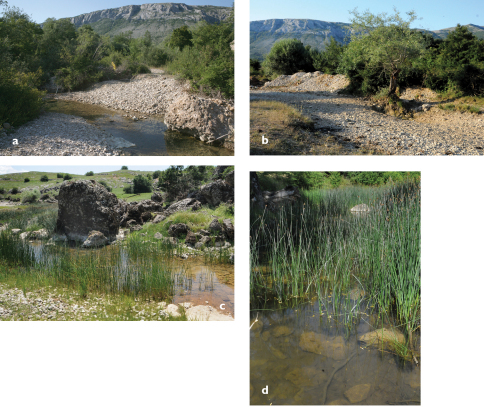
**a** Habitat of*Telestes dabar*: Opačica River at Potkom, Dabarsko Polje (type locality) **b** Opačica River 100 m away from the spring (8 July 2011) **c–d** habitat of *Telestes metohiensis*: Nevesinjsko Polje; **c** – spring Ljeskovik in Zalomka River (8 July 2011) **d** Zovidolka River at Udbine (8 July 2011). All in Bosnia and Herzegovina.

From May through September *Telestes dabar* is found in shallow water of those river sections that are adjacent to and filled from underground springs. There is no current, and the water is clean (Fig. 5a). Females with eggs and just-spent females were caught on 24 May 2001 in Vrijeka River and mature males and just-spent females on 31 May 2000 in Opačica River. The size of the ripe eggs was 1.3–1.7 mm in diameter. In all examined samples females predominate. The smallest spent female was 45.0 mm SL, and the smallest ripe male 43.7 mm SL. No other fishes were caught in Opačica together with *Telestes dabar* while *Delminichthys ghetaldii* were collected in Vrijeka.

#### Etymology.

The specific name, *dabar*, refers to the type locality, Dabarsko, or Dabar Polje; it is a noun in apposition.

### Telestes miloradi sp. n.

urn:lsid:zoobank.org:act:9A45C6CE-B7BF-4593-BAF6-0D10C45C78EA

http://species-id.net/wiki/Telestes_miloradi

Fig. 7

#### Holotype.

NMW 95296 (ex 51169), 66.7 mm SL; CROATIA: stream Ljuta at Gruda [misspelt Grinda in Steindachner (1901: 197)), 1901, coll. Kolombatovič.

#### Paratypes.

NMW 51169, 13 (? syntypes [now paralectotypes] of *Paraphoxinus metohiensis*), 31.4–62.6 mm SL, same data as holotype; NMW 51170, 4 (syntypes [now paralectotypes] of *Paraphoxinus metohiensis*), 57.9–66.4 mm SL, same data as holotype; NMW 51171, 3 syntypes [now paralectotypes] of *Paraphoxinus metohiensis*, 74.6–83.1 mm SL, same data as holotype; NMW 51173, 1 syntype [paralectotype] of *Paraphoxinus metohiensis*, 119.3 mm SL, same data as holotype.

#### Diagnosis.

*Telestes miloradi* is distinguished from *Telestes metohiensis* and *Telestes dabar* by having the following combination of characters: slightly curved, relatively narrow dark stripe (obvious in most preserved specimens) present on ventral portion of trunk from just behind operculum to vertical at or anterior to origin of anal fin, this stripe separated from dark pigmented area on back along its entire length; scales on most of body not overlapping; mouth subterminal with tip of mouth cleft at or below level of ventral margin of eye; snout not fleshy; lateral line complete with 55–67 total scales; branched dorsal-fin rays 8½; branched anal-fin rays 8½; gill rakers usually 8–10, mode 9; total vertebrae usually 40 or 41; abdominal vertebrae 22–23, mode 23; caudal vertebrae 16–18, mode 18; head width 48–58% HL, and lower jaw short, length 8–10% SL.

#### Description.

Morphometric data are summarised in Table 1b, selected counts in Tables 2–4. General appearance can be seen in Figs 7a and 7b. Body compressed, elongate. Caudal peduncle depth equal to or only slightly less than half maximum body depth; head length greater than maximum body depth. Eye small, its diameter smaller than snout length. Snout not fleshy, rostral cap covering only part of upper lip, at least in preserved specimens. Mouth subterminal, tip of mouth cleft at level of ventral margin of eye or, more frequently, below it. Lower jaw-quadrate junction at vertical through anterior half of eye. Length of lower jaw 8–10% SL or 33–38% HL, or 96–107% depth of operculum (equal to depth of operculum on average).

Dorsal fin with 8½ branched rays. Dorsal-fin origin above posterior end of pelvic-fin base. Anal fin with 8½ branched rays. Outer margin of anal fin slightly concave. Caudal fin moderately forked, lobes weakly pointed, with 9+8 principal branched rays. Total gill rakers (Table 2) 8 (1 specimen), 9 (11) or 10 (6), 10 in holotype. Pharyngeal teeth 5–4, hooked, slightly serrated (examined in 5 specimens).

Scales covering entire body including pre-pectoral area and abdomen, overlapping on most parts of body. Scales regularly set; lateral-line scales and scales above and below it of about equal size. Lateral line complete (Table 3), 55–67 scales in total, 61 in holotype. Lateral line not curving above anal-fin origin. Number of scales in total lateral series 58–67 (modal range 62–64), 63 in holotype.

Parietal segment of CSO lacking. CPM not communicating with CIO, terminating over upper margin of opercular antedorsal process or communicating with CIO (on one side in 3 specimens). CSO complete with 8, rarely 7 or 9, pores. CIO complete with 14–16 pores and with 4 canal openings on first infraorbital. CPM complete, with 14–16 pores (4 canal openings on dentary, and 7–9, usually 8, on preoperculum). CST complete, with 5 or 7 pores.

Total vertebrae (Table 4) 39 (1), 40 (10) or 41 (11), 41 in holotype; abdominal vertebrae 22 (8) or 23 (14), 23 in holotype; caudal vertebrae 17 (5), 18 (16) or 19 (1), 18 in holotype; predorsal vertebrae 13 (15) or 14 (7), 14 in holotype; intermediate vertebrae 3 (12) or 4 (9), 3 in holotype. Vertebral formulae 22+17 (1), 22+18 (7), 23+17 (5) and 23+18 (10), 23+18 in holotype.

#### Colouration.

In preserved specimens, dark back contrasting sharply with pale area below lateral midline. Dark midlateral stripe extending from head to caudal peduncle forming ventral border of darkly pigmented region on back(faded in some specimens). Another, more conspicuous, dark lateral stripe, occurring on ventral portion of trunk, narrow and not extending posterior to vertical through origin of anal fin. Peritoneum dark.

#### Sexual dimorphism

. Genital papilla absent in both males and females. Most morphometric characters not significantly different between males and females (Table 1). In the three male specimens examined, dorsal fin deeper than in females (P=0.0105); pectoral fin longer (P<0.001), pectoral fin nearly reaching pelvic-fin origin in males and well short of pelvic fin in females; and pelvic fin longer (P<0.0001), pelvic fin almost reaching anal-fin origin in males, well short of anal fin in females.

#### Distribution.

The new species is known from Ljuta River in Konavosko Polje, also called Konavoska Ljuta, of Dubrovnik littoral (Fig. 4). Only historical NMW samples are known to us.

#### Etymology.

The species is named for Milorad Mrakovčić, Zagreb, in recognition of his many contributions to the study of freshwater fishes in the Adriatic basin.

##### Comparative remarks

*Telestes dabar*, *Telestes miloradi*, *Telestes metohiensis*, *Telestes croaticus*, and *Telestes fontinalis* are distinguished from all congeners by having the pharyngeal teeth in one row, 5–4 or 5–5 (vs. usually 2.5–5.2 or 2.5–4.2), having the preoperculo-mandibular canal terminating in a free pore at the upper margin of the opercular antedorsal process and not communicating with the infraorbital canal (vs. communicating), and in lacking a postcleithrum (vs. postcleithrum present). All five species possess a dark midlateral stripe from the head to the caudal peduncle that forms the ventral border of the darkly pigmented area on the back. This feature is similar to that in some other *Telestes* species (Kottelat and Freyhof 2007: 282–289). Within this group, *Telestes dabar*, *Telestes miloradi*, and *Telestes metohiensis* differ from *Telestes croaticus* and *Telestes fontinalis* in having an additional black lateral stripe occurring on the otherwise pale ventral portion of the trunk. This character was considered unique for *Telestes metohiensis* (Kottelat and Freyhof 2007: 284). In *Telestes dabar* and *Telestes miloradi* this ventral stripe (Fig. 1a, 2a, 7) is narrow and extends from just behind the operculum maximally to a vertical through the origin of the anal fin. In *Telestes metohiensis*, the stripe (Fig. 1b, 2b–c) is wide and usually extends posteriorly to the caudal peduncle where it merges with the main pigmented area. The pale area between the dark area on the back and the ventral stripe varies in length and depth, being the smallest in females (Fig. 1b, 2b–c).

Besides the presence of the ventral stripe, *Telestes dabar* and *Telestes miloradi* are further distinguishable from *Telestes croaticus* and *Telestes fontinalis* by usually having 8½ branched dorsal-fin rays (vs. usually 7½). *Telestes dabar* differs from *Telestes croaticus* by usually having 40 total vertebrae (vs. usually 39) (Table 4); a maximum head width of 42–52% HL (averaging 50% HL in females and 48% HL in males), which is considerably smaller than the head depth at nape, 61–71% HL (averaging 67% HL in females and 65% HL in males) (vs. the maximum head width only slightly smaller than the head depth or about equal to it); and a smaller size, up to 82 mm SL (vs. up to 160 mm). *Telestes dabar* can befurther distinguished from *Telestes fontinalis* by having 5–4 pharyngeal teeth (vs. 5–5); a usually long, though slightly incomplete and narrowly interrupted, lateral line that reaches the posterior half of the caudal peduncle and has 24–69, usually 54–65, total scales (vs. a short, incomplete, and widely interrupted lateral line terminating in the area between the pectoral and anal fins with usually 23–37 total scales); usually 17 or 18 caudal vertebrae (vs. 16); usually 13 or 14 predorsal vertebrae (vs. 15) (Table 4); 3 or 4 intermediate vertebrae (vs. 5); and a moderately compressed body without any ventral keel (vs. a markedly laterally compressed body and a scaled ventral keel in front of the pelvic fins).

**Figure 6.**
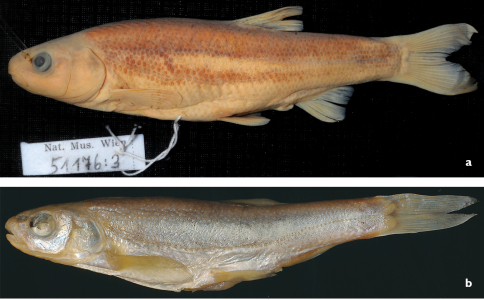
*Telestes metohiensis*. **a** NMW 51176:3, lectotype, 87.9 mm SL, ‘Mušica’ [Mušnica] River **b** NMW 51090 (labelled as *affinis* nomen museale), 57.9 mm SL, Zalomska [Zalomka River].

**Figure 7.**
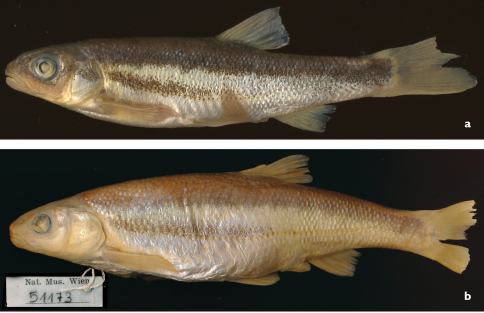
*Telestes miloradi*, Croatia: Ljuta River at Gruda, Konavosko Polje **a** Holotype, male, 66.7 mm SL, NMW 95296 **b** paratype, female, 119.3 mm SL, NMW 51173.

**Figure 8.**
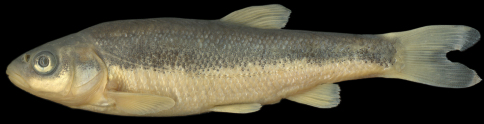
*Telestes karsticus*, PZC 504, 62.6 mm SL, Croatia: Sušik River, Danube drainage.

*Telestes miloradi* further differs from *Telestes croaticus* and *Telestes fontinalis* in usually having a long, complete lateral line with 55–67 scales vs. an often incomplete and interrupted lateral line with (18–45)51–70 and (17)23–37(56) scales, respectively. The new species further differs from *Telestes croaticus* in having 8–10, most frequently 9, gill rakers, (vs. 8–9, most frequently 8); usually 40 or 41 total vertebrae (vs. 38 or 39); 22–23, mode 23, abdominal vertebrae (vs. 21–23, mode 22); and 17–19, mode 18, caudal vertebrae (vs. 16–17, mode 17) (Table 4). *Telestes miloradi* can befurther distinguished from *Telestes fontinalis* by having 5–4 pharyngeal teeth (vs. 5–5); usually 40 or 41 total vertebrae (vs. 38–40, usually 39); 17–19, usually 18, caudal vertebrae (vs. 16–17, often 16) (Table 4); 3 or 4 intermediate vertebrae (vs. 5); and a moderately compressed body without a ventral keel (vs. a markedly laterally compressed body and a scaled ventral keel in front of the pelvic fins).

*Telestes miloradi* differs from *Telestes croaticus*, *Telestes fontinalis*, *Telestes metohiensis*, and *Telestes dabar* in having comparatively well-overlapped scales, especially on the anterior part of the flank and on the caudal peduncle; and scales of about the same size in the lateral line and above and below it (vs. scales usually non-overlapping on most of the body, and scales above and below the lateral line smaller than the lateral-line scales). The scale pattern in *Telestes miloradi* is very similar to the pattern found in most leuciscine fishes, e.g. *Telestes karsticus* Marčić & Mrakovčić, 2011 (Fig. 8), *Telestes turskyi* (Heckel, 1843), *Telestes ukliva* (Heckel, 1843). The presence of overlapping scales is a plesiomorphic feature for the Leuciscinae (Bogutskaya 1990, 1991), as is an interconnection of CPM and CIO (e.g., *Telestes karsticus*). In addition to overlapping scales, a few specimens of *Telestes miloradi* exhibit interconnected CPM and CIO.

In this study, we compared specimens of *Telestes metohiensis* from Gatačko, Cerničko, and Nevesinjsko poljes and found that specimens from the Nevesinjsko Polje (labeled as *affinis* by Steindachner although the name *affinis* was never published), though somewhat different in usually having smaller and more scattered scales, are similar to *Telestes metohiensis* from the Gatačko and Cerničko poljes in all other aspects examined by us (Tables 1c–d, 2–4). We suppose that a reason for Steindachner’s opinion could be the differences evident between the NMW specimens of *Telestes metohiensis* from the Gatačko Polje (Mušnica River, Gacko) and those from the Nevesinjsko Polje (Zalomka River) (Fig. 6). Additional material examined by us revealed that the differences noted above in scales may be size-dependent: larger specimens of *Telestes metohiensis* in both poljes usually have more densely set scales. A specimen of *Telestes metohiensis* from Gatačko Polje, NMW 51176:3 (87.9 mm SL) is designated here as lectotype to ensure taxonomic stability in the event that *Telestes metohiensis* from the Nevesinjsko Polje is recognised as taxonomically distinct in the future.

Both *Telestes dabar* and *Telestes miloradi* are distinguishable from *Telestes metohiensis* by usually having 8½ branched dorsal-fin rays (vs. usually 7½: 7½ found in 192 specimens and 8½ in 22); 39–41, modes 40 and 41, total vertebrae (vs. 38–40, rarely 41, mode 39); 16–19, modes 17 and 18, caudal vertebrae (vs. 15–17, usually 16 or 17) (vs. 15–17, usually 16 or 17) (Table 4; differences are statistically significant at P<0.0001); and more numerous gill rakers, 9 or 10 (vs. (7)8–9(10), most frequently 8) (Table 2). *Telestes dabar* further differs from *Telestes metohiensis* in usually having an interrupted lateral line with 24–69, usually 54–65, total scales (vs. usually complete with 58–65 scales) (Table 3; difference is statistically significant at P<0.0001). The scale pattern also distinguishes *Telestes dabar* from *Telestes metohiensis*. In *Telestes dabar*, the scales (Fig. 3a) are densely set, but they do not overlap on most parts of the body except behind the pectoral girdle and on the caudal peduncle; the scales above and below the lateral line are only slightly smaller than the lateral-line scales; and the scales are oval, somewhat deeper than long, on the flanks and elongated, longer than deep with a prominent posterior attenuation, on the caudal peduncle. In *Telestes metohiensis* the scales are more or less widely spaced and do not overlap on the entire body except for the lateral line; this feature can be seen in live specimens, Fig. 2b); the scales (Fig. 3b) above and below the lateral line usually are considerably smaller than the lateral-line scales; and the scales are almost circular on both the flanks and the caudal peduncle. *Telestes dabar* is further distinguished from *Telestes metohiensis* by having a black-and-white general colouration except for yellowish-orange pigmentation of the fin bases and yellow pigmentation in the iris and along the operculum in adults (vs. yellowish-green or greenish-bronze pigmentation of the whole body and fins in both young and adults, Fig. 2b–c). With regard to the morphometric features, *Telestes dabar* is rather similar to *Telestes metohiensis*,differing only in having a narrower head, maximum head width 12–15% SL or 43–52% HL and maximum cranial width 60–73% cranium roof length (vs. 13–17% SL or 51–59% HL, and 64–80% cranium roof length) (Table 1; differences are statistically significant at P<0.0001).

Besides the characters mentioned above, *Telestes dabar* differs from *Telestes miloradi* in having the lateral-line scales larger than the scales above and below it (vs. of about equal size); usually an interrupted lateral line making a sharp curvature upward above the anal-fin base (vs. complete and making no sharp curvature); the length of lower jaw 10–12% SL, 36–41% HL, 102–132% depth of operculum (vs. 8–10% SL, 33–38% HL, 96–107% depth of operculum) (Table 1; differences are statistically significant at P<0.0001).

##### Comments on the distribution and conservation of *Telestes dabar*, *Telestes miloradi* and *Telestes metohiensis*

The three *Telestes* species are known from four of the 13 main karst poljes located in Eastern Herzegovina (Bosnia and Herzegovina) and in Dubrovnik littoral area (Croatia) (Fig. 4). These karst poljes are part of a high-karst geotectonic unit known as Dinaric Karst, which consists of Mesozoic carbonate formations. The depth of soluble and highly karstified rocks here exceeds 3000 m (Milanović 1981, 2006). A polje (means “field” in many Slavic languages) is a large closed depression draining underground with a flat floor. Its streams may be permanent, intermittent and perennial, and, in natural conditions, a polje is subject to periodic flooding and becomes a lake. In Eastern Herzegovina and in the Dubrovnik littoral area stepwise poljes are distributed from 60 m up to 1,080 m above sea level (Fig. 4). Streams and rivers appear from temporary or permanent springs and sink underground through swallow holes called ponors. In general, hydro-systems of all poljes under consideration (Fig. 4), except for the Konavosko Polje with direct connection to the Adriatic Sea and the Polje Gradac with no springs or surface flows, belong to the Neretva drainage area and form a complex net of underground flows. Within the Neretva drainage, the poljes belong to catchments of the Buna River (Slato and Nevesinjsko), the Bregava River (Lukavačko and Dabarsko), and the Trebišnijca River (Gatačko, Fatničko and others). At present, no one polje has a direct groundwater (surface) flow connection with Neretva or its tributaries. Historically, interconnections between variable surface streams and between them and the main Neretva course occurred during different geological epochs. Fish distributions can provide some evidence of this. Conversely, very local distribution of some fishes may indicate the isolation of some surface drainage systems for a long time.

Slato [Zlato] Polje (1,020‒1,080 m above sea level) is situated at the highest elevation of all Eastern Herzegovina poljes. Ćurčić (1915a) reported finding no fishes at Slato Polje, and no fishes are known from later literature or from museum collections. The Slato Polje is connected with Nevesinjsko Polje through a narrow valley that is now dry. The Nevesinjsko Polje, the largest polje in Eastern Herzegovina, has a surface area of 170 km^2^ and is located at an elevation from 870 m to 800 m above sea level. The lowest point is Biograd Ponor, which is the terminus of the Zalomka River that starts at Raščelica near the Gatačko Polje. This river has a permanent flow only between Fojnica and Črni Kuk. Along the river bed downstream from Črni Kuk there are a lot of ponors. The most prominent leakage zone is in the Rilja section where the Zalomka is active only 213 days per year, on average (Milanović 1981, 2006). In the warm season almost the entire river bed of the Zalomka within the Nevesinjsko Polje is dry, and fishes are found in its upper section only. Only four species are known from there: *Salmo* sp., *Squalius* cf. *squalus*, *Squalius svallize* Heckel & Kner, 1858, *Phoxinus* sp. (PZ personal observations). Ćurčić (1915a) reported that *Paraphoxinus metohienis* was the most numerous species around Fojnica, but at present only *Phoxinus* sp. was found there by PZ. Further downstream, *Telestes metohiensis* occurs in those very short river sections that are adjacent to permanent springs such as Ljeskovik. This species also occurs in upper reaches of the Zavidolka River that temporarily flows to the Zalomka in the east from the Biograd Ponor (our data). *Delminichthys ghetaldii* is absent from the Nevesinjsko Polje and the Zalomka system.

Dabarsko Polje, about 20 km long and 1 to 3 km wide, is located close to the Nevesinjsko Polje but isolated from it. The Dabarsko Polje lies more than 400 m of elevation below the Nevesinjsko Polje. At present, the Dabarsko Polje is a closed basin without a possibility for surface runoff. All waters of the Dabarsko Polje catchment flow through underground karst conduits toward the springs of the Bregava River, though historically the Polje drained to the Bregava River canyon that is now dry. The lowest point is the Ponikva Ponor (471 m above sea level), the terminus of a single permanent stream in the polje, the Vrijeka River, which is only 2.5 km long (Milanović 1981, 2006). The Opačica River located in the northwestern part of the polje is longer but intermittent. Only two native species occur in Dabarsko Polje: *Delminichthys ghetaldii* and *Telestes dabar* (recorded earlier under the name *Phoxinellus metohiensis*) in Vrijeka and only *Telestes dabar* in Opačica (our data). *Delminichthys* (as *Phoxinellus*) *ghetaldii* was first recorded in Sušica and Ljelješnica cave springs by Ćurčić (1915a) and then confirmed by PZ’s findings in a stream flowing from the Ljelješnica Cave (Zupančič and Bogutskaya 2002) and in the Vrijeka. It is relatively less abundant in Vrijeka than *Telestes dabar*. A trout has been introduced into Vrijeka.

The small Lukavačko Polje (2.5 km^2^) is located at an elevation of 852–880 m east from the Dabarsko Polje. The two poljes are divided by about 400 m of elevation. Fatničko Polje is also a small closed basin (5.6 km^2^) located southeast from the Dabarsko Polje at a much lower elevation, 462–470 m above sea level. The Fatničko Polje is divided from the Dabarsko Polje by an extremely karstified limestone ridge which is about 2 km wide. The most prominent karst features of the Fatničko Polje are the Obod temporary spring and the Pasmica Ponor. About 85% to 90% of the Fatnicko Polje water flows to the Trebišnjica springs and 10% to 15% to the Bregava springs (Milanović 1981, 2006). Ćurčić (1915a, 1915b), Taler (1953a, 1953b), Sabioncello (1967) and Vuković (1977) reported *Paraphoxinus metohiensis* from the Lukavačko and Fatničko poljes. However, no extant samples confirm these reports. In 1998–2001, PZ found only *Delminichthys ghetaldii* in Fatničko Polje and no *Delminichthys ghetaldii* or *Telestes metohiensis* in the Lukavačko Polje (Zupančič and Bogutskaya 2002). No other data have been received since then.

Gatačko Polje (37.6 km^2^) consists of two geomorphologically and hydrogeologically interconnected units: Gatačko Polje itself and Small Gatačko Polje. The largest ponor zone is situated in Small Gatačko Polje along the 8 km long tectonic contact between flysch sediments and karstified limestone, from Srdevići to the Šabanov Ponor (936 m above sea level). The entire Gatačko Polje belongs to the catchment area of the Trebisnjica River, except a very small eastern part. The longest underground flow (35 km) in Eastern Herzegovina is between the Srdevići Ponor and the Trebisnjica Springs. The main flow in the Gatačko Polje is the Mušnica River with its tributary Gračanica. The Mušnica is formed by three streams, Vrba, Ulinjski Potok and Jasenički Potok. They flow from the Cemern and Lebršnik mountains. The Mušnica goes from the eastern to the western border of the polje and along its western border southwards before it completely sinks in the Šabanov Ponor. The flow of the Mušnica River re-appears in the Cerničko Polje where it is named the Ključka River. It originates in a large cave, Vilina Pećina and terminates about 300 m downstream in a ponor that has a recharge capacity of approximately 20 m^3^ s^−l^ (Milanović 2006). *Telestes metohiensis* (in *Paraphoxinus* or *Phoxinellus* by earlier authors), *Phoxinus* sp. and *Alburnus neretvae* Buj, Sanda et Perea 2010 are known to occur in Mušnica. *Phoxinus* sp. and, probably, *Salmo* sp. occur in Gračanica. Many literature sources (see in Zupančič and Bogutskaya 2002) reported *Paraphoxinus metohiensis* from the Gračanica River at Gacko, but we know of no extant samples. Only *Telestes metohiensis* was found (PZC) in short karstic streams of the Cerničko Polje.

*Telestes metohiensis* or close species are absent from other poljes except for the Konavosko Polje. This polje is located rather far in the south from poljes inhabited by *Telestes metohiensis* and *Telestes dabar*. The Konavosko Polje or Polje Konavliis the lowermost polje in the region (elevation 60 m above sea level) with surface of 48 km^2^. It is developed along the most important overthrust of the entire dinaric karst region (“High Karst Overthrust”). The largest spring Konavoska Ljuta is located at this contact. In natural conditions it was a temporary flooded polje. Main flows are Ljuta and its tributaries Konavočica and Opačica. At present, a tunnel between the polje and the sea coast drains the polje (Milanović 2006). No recent records exist of a *Telestes* species in the Ljuta or other springs of the Konavosko Polje. However, we think that efforts are worth trying to find *Telestes miloradi*, a new species, described here from the Konavoska Ljuta before it is finally considered extinct. It is known that many species from *Phoxinellus*, *Telestes* and *Delminichthys* genera are able to enter karstic underground waters during droughts and dramatic water table level fluctuation (Milanović 2006; Jelić, 2008; Marčić et al. 2011). However, this phenomenon probably depends considerably on the degree of development, size and depth of caverns, siphonic lakes and pools of estavelles.

The ranges of *Telestes metohiensis* and *Telestes dabar* are extremely small at least during the dry season and in surface water bodies. The recognition of *Telestes dabar* as a distinct species will require its Red List evaluation and a re-evaluation of *Telestes metohiensis* (now considered as Vulnerable) because of the loss of a part of its former presumed range. *Telestes dabar* would probably deserve the Critically Endangered status because of its very restricted distribution (IUCN 2010). Both species are threatened by habitat destruction, in particular by construction of tunnels for the draining of poljes and controlling their inundations, lining of river beds to obstruct water flow into ponors and reversible communication with estavelles.

##### Key to *Telestes* species of isolated karst river systems of the Adriatic basin in Bosnia and Herzegovina and Croatia, including Krbavsko Polje

**Table:** 

1a	Pharyngeal teeth in two rows, usually 2.5–5.2 or 2.5–4.2. Preoperculo-mandibular canal communicating with infraorbital canal. Postcleithrum present, of moderate size	2
1b	Pharyngeal teeth in one row, usually 5–4. Preoperculo-mandibular canal not communicating with infraorbital canal. Postcleithrum minute or absent	3
2a	Branched dorsal-fin rays 8½ and branched anal-fin rays 9½. Total lateral-line scales 60–68	*Telestes ukliva*
2b	Branched dorsal-fin rays 7½ and branched anal-fin rays 8½. Total lateral-line scales 72–79	*Telestes turskyi*
3a	Black stripe on ventral portion of trunk narrow and not reaching caudal peduncle. Branched dorsal-fin rays usually 8½	4
3b	Black stripe on ventral portion of trunk absent or present; if present, stripe wide anteriorly and extending posteriorly to caudal peduncle. Branched dorsal-fin rays usually 7½	5
4a	Scales overlapping on most of body, scales approximately same size in, above and below lateral line. Lateral line not curving above anal-fin origin. Lower-jaw length 8–10% SL	*Telestes miloradi*
4b	Scales non-overlapping on most of body, though densely set. Lateral-line scales larger than scales above and below lateral line. Lateral line making clear curvature above anal-fin origin. Lower-jaw length 10–12% SL	*Telestes dabar*
5a	Body strongly compressed, abdomen with weakly scaled ventral keel in front of pelvics. Lateral line short, incomplete, terminating between pectoral and anal fins, and usually widely interrupted, with 17–56, usually 23–37, total scales. Branched anal-fin rays 7½	*Telestes fontinalis*
5b	Body slightly compressed, abdomen rounded. Lateral line long, slightly incomplete or complete, usually terminating on caudal peduncle, and narrowly interrupted, with 24–70, usually 54–68, total scales. Branched anal-fin rays usually 8½	6
6a	Black stripe on ventral portion of trunk present	*Telestes metohiensis*
6b	Black stripe on vental portion of trunk absent	*Telestes croaticus*

##### Comparative material

*Telestes karsticus*. PZC 577, 7, 547–750 mm SL, CROATIA: Sušik River at Tomići, Dobra River system (Danube drainage), 24 June 2005, coll. Zupančič.

*Telestes metohiensis*. All from BOSNIA AND HERZEGOVINA. **Gatačko Polje:** NMW 51176:3, lectotype, mm SL, 87.9 mm SL, ‘Mušica bei Imotski’ [misspelling, should be Mušnica River, Gatačko Polje; Imotski is probably an error because Steindachner (1901: 198) clearly indicated that the specimens had been received from karst waters and streams near Gacko, a town in the north of Gatačko Polje (43°09.4'N, 18°31.8'E); see also Zupančič and Bogutskaya 2002], 1899, coll. Redel, Sturani [Sturany]; NMW 51176:1–2, 72.2–82.5 mm SL, same data as holotype; NMW 12972, 1 paralectotype, 92.2 mm SL, same data as holotype; NMW 12973–75 (in one jar with 12972), 3 paralectotypes, 59.2–95.3 mm SL, same data as holotype; NMW 51172, 2 paralectotypes, 92.0–97.8 mm SL, same data as holotype; NMW 51174, 2 paralectotypes, 76.4–88.5 mm SL, same data as holotype; NMW 51175, 3 paralectotypes, 66.8–75.9 mm SL, same data as holotype; SMF 805, 2, 53.2–65.6 mm SL, Gacko; ZMH 15137, 7, 51.2–64.4 mm SL, Gacko, Herzegovina; **Cerničko Polje:** PZC 223, 9, 45.0–90.6 mm SL, source of Ključka River in Vilina Pečina (cave), 43°5.6'N, 18°29'E, 16 May 2003, coll. Zupančič; PZC 330, 9, 46.0–88.7 mm SL, same locality and collector, 23 May 2001; PZC 337, 4, 73.7–85.4 mm SL, same locality and collector, 20 Aug. 2000; PZC 368, 4, 49.0–62.2 mm SL, spring at Ključ, 43°5.6'N, 18°29.6'E, 23 May 2000, coll. Zupančič; PZC 503, 7, same locality and collector, 3 Aug. 2000. **Nevesinjsko Polje:** (NMW syntypes [now paralectotypes] of *Paraphoxinus metohiensis* labeled as *Paraphoxinus affinis* [handwritten by Steindachner; never published], nomen museale) NMW 9368–9372, 5, Zalomska [Zalomka River], 1896, coll. Hawelka; NMW 51088, 10, 47.6–57.9 mm SL, same data as above; NMW 51089, 10, 49.6–58.1 mm SL, same data as above; NMW 51092, 5, 51.9–65.5 mm SL, same data as above; NMW 51093, 8, 47.4–58.7 mm SL, same data as above; NMW 51094, 10, same data as above; NMW 51090, 5 (not labeled as syntypes of *Paraphoxinus metohiensis* though exclusion of these specimens from the type series is not evident from the original description), 47.6–57.9 mm SL, same data as above; NMW 51091, 4 (not labeled as syntypes, as above), 63.5–87.1 mm SL, same data as above; PZC 206, 3, 50.4–73.6 mm SL, Batuša River (Zalomka system), 43°19.3'N, 18°7.1'E, 9 May 2000, coll. Zupančič; PZC 355, 17, 39.6–63.8 mm SL, same locality and collector, 21 May 2001; PZC 356, 6, 53.2–66.1 mm SL, same locality and collector, 5 May 2000; PZC 312, 19, 50.1–79.8 mm SL, spring Ljeskovik in Zalomka River near Rast and Odžak, 43°12.1'N, 18°12.4'E, 21 May 2001, coll. Zupančič; PZC 313, 14, 49.1–78.7 mm SL, same locality and collector, 1 July 2002; PZC 567, 7, 45.1–86.2 mm SL, same locality and collector, 8 July 2011; PZC 358, 2, 38.2–42.0 mm SL, spring in Zalomka River at Budisavlje, 43°13.3'N, 18°13.1'E, 22 May 2011, coll. Zupančič; PZC 523, 15, 39.5–90.9 mm SL, Zovidolka [Zavodoka, Zovidolska] River at Udbine, 43°8.6'N, 18°14.7'E, 15 Sept. 2006, coll. Zupančič; PZC 293, 12, 49.1–99.2 mm SL, Zovidolka [Zavodoka, Zovidolska] River at Udbine, 43°8.6'N, 18°14.7'E, 29 Aug. 2006, coll. Zupančič; PZC 315, 17, 57.8–81.4 mm SL, same locality and collector, 16 July 2002; PZC 523, 15, 39.5–90.9 mm SL, same locality and collector, 15 Sept. 2006; PZC 524, 13, 39.5–90.9 mm SL, same locality and collector, 15 Sept. 2006; PZC 566, 45, same locality and collector, 8 July 2011.

*Telestes polylepis*. NMW 18931–41, 11, paralectotypes, 93.6–107.2 mm SL, CROATIA: Josefsthal [Josipdol], 1866; NMW 49713, 3, syntypes, 86.7–90.7 mm SL, CROATIA: Dobra River, 1866; NMW 49714–1, lectotype, 85.9 mm SL, CROATIA: Mresnitza [Mrežnica] River, 1866; NMW 49715, 2, paralectotypes, 80.8–84.1 mm SL, same data as lectotype.

*Telestes turskyi*. NMW 49629-1, lectotype, 129.5 mm SL, CROATIA: Čicola River [tributary of Krka], Dernis [Drniš] “Heckels Reise, 1840”; 17, paralectotypes, 55.3–102.3 mm SL.

*Telestes ukliva*. NMW 49639-1, lectotype, 85.0 mm SL, CROATIA: Sign “[aus der Cettina]”, “Heckels Reise, 1840”; NMW 49639-2 and 3, 2, 83.3–84.8 mm SL, same data as lectotype; NMW 49635, 3, paralectotypes, 58.0–75.8 mm SL, same data as lectotype; ZMH 15095, 1, CROATIA: Cetina.

Other extensive comparative material is listed in Zupančič and Bogutskaya (2002) and Bogutskaya and Zupančič (2003).

## Supplementary Material

XML Treatment for Telestes dabar

XML Treatment for Telestes miloradi
